# Fortified Extract of Red Berry, *Ginkgo biloba*, and White Willow Bark in Experimental Early Diabetic Retinopathy

**DOI:** 10.1155/2013/432695

**Published:** 2013-05-23

**Authors:** Claudio Bucolo, Giuseppina Marrazzo, Chiara Bianca Maria Platania, Filippo Drago, Gian Marco Leggio, Salvatore Salomone

**Affiliations:** Department of Clinical and Molecular Biomedicine, Section of Pharmacology and Biochemistry, University of Catania, Viale Andrea Doria 6, 95125 Catania, Italy

## Abstract

Diabetic retinopathy is a complex condition where inflammation and oxidative stress represent crucial pathways in the pathogenesis of the disease. Aim of the study was to investigate the effects of a fortified extract of red berries, *Ginkgo biloba* and white willow bark containing carnosine and *α*-lipoic acid in early retinal and plasma changes of streptozotocin-induced diabetic rats. Diabetes was induced by a single streptozotocin injection in Sprague Dawley rats. Diabetics and nondiabetic (control) rats were treated daily with the fortified extract for the ten days. Retina samples were collected and analyzed for their TNF-*α* and VEGF content. Moreover, plasma oxidative stress was evaluated by thiobarbituric acid reacting substances (TBARS). Increased TNF-*α* and VEGF levels were observed in the retina of diabetic rats. Treatment with the fortified extract significantly lowered retinal cytokine levels and suppressed diabetes-related lipid peroxidation. These data demonstrate that the fortified extract attenuates the degree of retinal inflammation and plasma lipid peroxidation preserving the retina in early diabetic rats.

## 1. Introduction

Diabetic retinopathy, a diabetes-related complication, is the leading cause of blindness and visual impairment in working-age individuals [1]. Diabetic retinopathy is a chronic disease that develops in stages and is rarely detected in the first few years of diabetes. The incidence of the disease increases to 50% by 10 years and to 90% by 25 years of diabetes [1].

Oxidative stress appears to be an important feature of the diabetic complications such as retinopathy. Apart from the well-known increase in lipid peroxide, diabetics have lower concentrations of erythrocyte glutathione and have higher concentrations of dehydroascorbate in their plasma and lower levels of vitamin E in their platelets. Oxidative stress causes a production of chemically reactive molecules, which induce a variety of proinflammatory mediators such as VEGF and TNF-*α* [2, 3]. The earliest changes detectable in diabetic retinopathy are loss of pericytes, capillary basement membrane thickening, edema, and formation of microaneurysms. These structural and functional changes are followed by microvascular occlusion, neovascularization, and neurodegeneration [4].

Considering that oxidative stress and inflammation represent the key factors in the onset and progression of diabetic retinopathy, antioxidant and anti-inflammatory products are expected to produce significant therapeutic advantages.

Current treatments associated with antidiabetic drugs are mostly intended to regulate vascular changes, inflammation, and the increased oxidative stress. Dietary supplements have been shown to play an important role in ameliorating clinical signs of diabetes [5]. Many studies have identified flavonoids that are associated with a reduction in the risk of advanced retinal degeneration. Recently [6], it has been demonstrated that eriodictyol, a strong antioxidative flavonoid extracted from *Eriodictyon californicum*, significantly reduces the retinal levels of VEGF, ICAM-1, TNF-*α*, and eNOS in diabetic rats.

Much evidence supports the roles of berry extracts, *α*-lipoic acid, and L-carnosine as antioxidant and anti-inflammatory products [5, 7, 8]. It has been demonstrated that supplementation with these compounds can inhibit retinal diabetes-induced abnormalities [5]. In particular, *α*-lipoic acid can scavenge free radicals and can act as an antioxidant [9]. Alpha-lipoic acid acts both directly, by radical quenching and metal chelation, and indirectly through the recycling of other antioxidants such as ascorbic acid, vitamin E, and glutathione [9]. Alpha-lipoic acid has been shown to protect against cataract formation [10, 11], a diabetic complication resulting from polyol accumulation, and it is beneficial also in a rat model of diabetic retinopathy [12]. Alpha-lipoic acid attenuates apoptosis in the retinal capillary endothelial cells of rats and decreases the levels of oxidative stress markers such as 8-hydroxydeoxyguanosine (8-OHdG) and nitrotyrosine [13]. Furthermore, Kowluru et al. [14, 15] demonstrated that its supplementation completely prevents the diabetes-induced increase in nitrotyrosine and activation of NF-*κ*B, while decreasing the levels of VEGF in the rat retina [14, 15]. Multiple biochemical pathways that are known to increase the production of reactive oxygen species (ROS), advanced glycosylation end products (AGEs), and reactive nitrogen species (RNS) have been linked to hyperglycemia/diabetes-induced vascular injury [16].

L-Carnosine, a dipeptide primarily produced in skeletal muscle and the central nervous system, acts both as a scavenger of ROS and as an inhibitor of AGE production [8]. L-carnosine is one of the most abundant antioxidants in the brain and retina [8]. Carnosine is synthesized from *β*-alanine and histidine and is specifically degraded by selective enzymes such as carnosinase-1 (CN-1). It has been suggested that the carnosine/carnosinase system plays an important role in the pathogenesis of diabetic complications [8]. Red berries are rich in phenolic compounds as well as many other essential nutritional components, such as flavonoids and phenolic acids, which have a wide range of beneficial properties, including retinal protection [17]. Epidemiologic evidence suggests that a high consumption of flavonoids may be useful against coronary heart disease, stroke, and neurodegenerative disorders [18, 19]. The high level of scavenging activity of red berry extracts toward chemically generated reactive oxygen species has been described in several studies [18]. *Salix alba* (white willow bark) extract is used for anti-inflammatory medical treatments due to its ability to suppress prostaglandin synthesis. The main component of *Salix alba* is salicin, an analogue of the widely used acetyl salicylic acid [20]. Two trials investigating the effects of *Salix alba* found evidence that daily doses standardized to 120 mg or 240 mg of salicin were better than placebo for short-term improvements in pain and rescue medication [20, 21].

*Ginkgo biloba* leaf extract (GBE) contains many different flavone glycosides and terpenoids [22]. It is well known that GBE has an antioxidant action as a free radical scavenger, and an anti-inflammatory effect suppressing the production of active oxygen and nitrogen species [22]. GBE inhibits the increase in the products of the oxidative decomposition of low-density lipoprotein (LDL), reduces the cell death in various types of neuropathy, and prevents the oxidative damage to mitochondria, suggesting that its beneficial effects on neurodegenerative diseases are related to prevention of chronic oxidative damage [23]. 

In the present study, we investigated the effect of systemic treatment with a fortified extract (FE) on proinflammatory mediators (TNF-*α* and VEGF), in the diabetic rat retina. Moreover, we evaluated plasma oxidative stress by measuring the thiobarbituric acid reacting substances (TBARS) [24]. 

## 2. Materials and Methods

### 2.1. Animals and Reagents

Male Sprague Dawley rats (approximately 200 g) were obtained from Charles River (Calco, Italy). All the animals were treated according to the ARVO Statement for the Use of Animals in Ophthalmic and Vision Research and the Directive 2010/63/EU of the European Parliament and of the Council. The animals were fed on standard laboratory food and were allowed free access to water in an air-conditioned room with a 12 h light/12 h dark cycle. Final group sizes for all measurements were *n* = 8–10. STZ was purchased from Sigma-Aldrich (St. Louis, MO, USA). All other reagents were purchased from standard commercial suppliers unless otherwise noted.

### 2.2. Induction of Diabetes and Treatment Schedule

STZ acts by producing concentrations of peroxides greater than can be tolerated by the islets of Langerhans, since these are poor in glutathione peroxidase. The induction of diabetes was performed as previously described [24]. Briefly, the animals received a single injection (iv) of STZ (60 mg/kg). Control (nondiabetic) animals received the vehicle alone. After 24 h, animals with blood glucose levels greater than 250 mg/dL were considered diabetic and randomly divided into groups. All experiments were carried out 10 days after induction of diabetes. We confirmed the diabetic state by evaluating glycemia daily using a blood glucose meter (Accu-Check Active; Roche Diagnostic, Milan, Italy). A group of rats were treated with the FE extract intraperitoneally (i.p.) starting from 30 min after STZ administration. Treatment consisted of daily injections with the blend suspension containing the following: 300 mg/kg *α*-lipoic acid, 150 mg/kg *Salix alba* extract (containing 15% salicin), 100 mg/kg berry extract (35% polyphenols; 6% anthocyanins), and 65 mg/kg *Ginkgo biloba* extract (22.0–27.0% *Ginkgo* flavonoids; 5.0–7.0% terpene lactones; ginkgolic acid content <5 ppm), and 50 mg/kg L-carnosine (Tiomax, Sooft Italia SpA, Montegiorgio, Italy). We chose these concentrations because they represent the dose recommended by an ophthalmologist in clinical practice [25]. After 10 days animals were killed, and retina and blood samples were collected to assess cytokines (TNF-*α* and VEGF) and TBARS, respectively.

### 2.3. Measurements of TNF-*α* and VEGF

After 10 days from the STZ injection, the eyes were enucleated and retinal samples were collected; each retina was handled as previously described [24]. Briefly, the retinal samples were homogenized in 100 *μ*L of cocktail solution supplemented with protease inhibitors before use. Samples were centrifuged, and protein levels were measured (Mini BCA Kit; Pierce Scientific, CA, USA). TNF-*α* and VEGF protein levels were estimated with commercial ELISA kits. The tissue sample concentration was calculated from a standard curve and corrected for protein concentration.

### 2.4. Lipid Peroxidation Assay

Lipid peroxidation is defined as “the oxidative deterioration of polyunsaturated lipids,” that is, lipids that contain more than two carbon-carbon double covalent bonds. As previously described [6], plasma lipid peroxidation was assessed by the thiobarbituric acid reacting substances method. The thiobarbituric acid test is one of the most frequently used tests for measuring the peroxidation of fatty acids. Briefly, plasma was mixed with hydrochloric acid and thiobarbituric acid, incubated and heated for 20 min, and then deproteinated with trichloroacetic acid. The absorbance of the malonaldehyde and thiobarbituric acid pink product was detected at 532 nm. The results are expressed in nmol MDA per mL of plasma.

## 3. Results 

### 3.1. Glycemia and Body Weight

Ten days after onset of diabetes, blood glucose values in diabetic rats treated with FE were significantly higher than corresponding values in nondiabetic rats (Table 1). FE does not interfere with glycemia values in nondiabetic rats (data not shown). Body weights of diabetic rats treated with FE were significantly less than those of nondiabetic rats but were not different compared with the diabetic group (Table 1). 

### 3.2. TNF-*α* and VEGF

**Figure 1:**
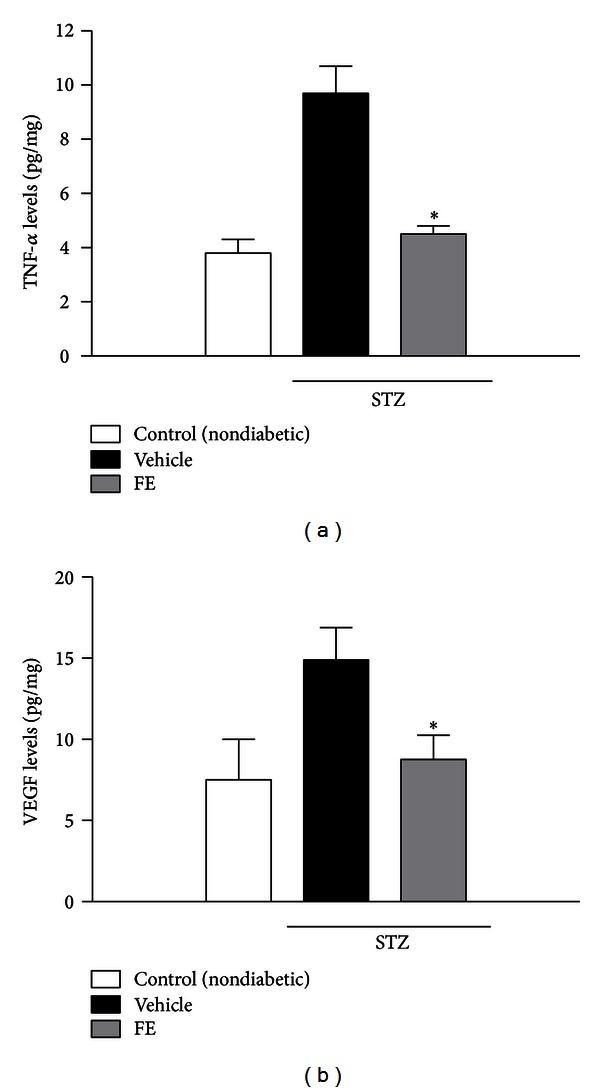
Retinal levels of TNF-*α* (a) and VEGF (b) 10 days after STZ injection with or without FE treatment. Data are expressed as the mean ± SD. *P < 0. 001 versus vehicle (*n* = 8–10).

Figure 1 shows the retinal TNF-*α* and VEGF levels. Experimental diabetes significantly increases the TNF-*α* level (from 3.8 ± 0.5 pg/mg to 9.7 ± 1.0 pg/mg; *P* < 0.001). FE treatment significantly reduced the retinal levels of TNF-*α* in the STZ-treated group (from 9.7 ± 1.0 pg/mg to 4.5 ± 0.3 pg/mg; *P* < 0.001). We also assessed the effect of FE treatment on VEGF content. As shown in Figure 1, STZ-treated animals showed a 2-fold increase in VEGF levels (from 7.5 ± 2.5 pg/mg to 14.9 ± 2.0 pg/mg; *P* < 0.001). FE treatment in diabetic rats significantly lowered VEGF levels compared with control animals (from 14.9 ± 2.0 pg/mg to 8.7 ± 1.5 pg/mg; *P* < 0.001). The FE did not significantly affect cytokine levels in normal nondiabetic rats (data not shown).

### 3.3. Lipid Peroxidation

**Figure 2:**
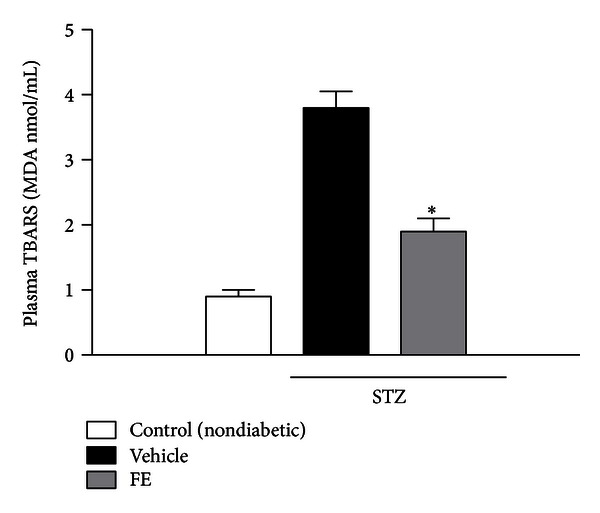
Lipid peroxidation. Effects of FE treatment in STZ-induced diabetic rats on the formation of plasma thiobarbituric acid reactive substances (TBARS). Data are expressed as the mean ± SD. *P < 0.001 versus vehicle (*n* = 8–10).

A significant increase in plasma lipid peroxidation was observed after 10 days of diabetes (Figure 2). FE treatment significantly (*P* < 0.001) suppressed diabetes-related lipid peroxidation (1.9 ± 0.2 MDA nmol/mL and 3.8 ± 0.1 MDA nmol/mL, resp.). The FE did not significantly affect lipid peroxidation in normal nondiabetic rats (data not shown).

## 4. Discussion

The present results show that FE of red berry, *Ginkgo biloba* and white willow bark, containing *α*-lipoic acid and L-carnosine, may blunt some of the negative effects due to hyperglycemia, such as oxidation, inflammation and VEGF expression, which are the main causes of diabetic retinopathy. 

Diabetes is characterized by a progressive vascular impairment mediated by pericyte loss that leads to an increase of retinal leakage and macular edema and the formation of new vessels [26]. Many studies demonstrated that VEGF and TNF-*α* contribute to the progression of diabetic retinopathy and that their expression is increased in the diabetic retina. The role of VEGF in the development of diabetic complications in the eye is well established, whereas the role of TNF-*α* is still under investigation. TNF-*α* has been implicated in the pathogenesis of diabetic retinopathy, this proinflammatory cytokine induces expression of endothelial adhesion molecules via activation of NF-*κ*B. This latter is able to increase the expression of other inflammatory mediators such as cyclooxygenase enzyme-2 (COX-2). This enzyme may also be activated by glycosylation products [27]. In the present study, we observed that TNF-*α* levels in the diabetic rat retina were significantly higher in comparison with retina obtained from the control group (Figure 1(a)). According to our data, other groups demonstrated that retinal TNF-*α* is significantly elevated in diabetic rats [28, 29]. TNF-*α* is a proinflammatory cytokine mainly generated by inflammatory cells and activated endothelial cells. This cytokine is a potent inducer of the leukostasis elicited by other actors such as VEGF, IL-1*β*, and PAF in the retinal vasculature [30]. As reported by Joussen et al. [31] TNF-*α* is one of the most important cytokines in diabetic retinopathy both involved in leukocyte activation and in endothelial cell apoptosis. Further, as showed by the same authors [29, 31], TNF-*α* receptor inhibitor and nonsteroidal anti-inflammatory drugs attenuated leukocyte adhesion in diabetic retinal vessels as well as the retinal leakage, suggesting a key role of TNF-*α*.

Our results, beside confirming that TNF-*α* levels are increased in the retina of STZ-induced diabetic rats, show that FE treatment prevents the increase in TNF-*α* levels (Figure 1(a)), suggesting that the antioxidant treatment improved the inflammatory milieu of the diabetic retina.

To further investigate whether this FE had any effect on VEGF levels in our model of early diabetic retinopathy, we also measured VEGF levels in retina.

VEGF is a growth factor, which stimulates angiogenesis, promotes vascular permeability, and induces dissociation of tight junctions. Production of VEGF is elicited by high glucose levels, AGEs, IGF-I, angiotensin II, and hypoxia; all these factors increase in the retinal diabetic microvascular bed [32]. Moreover, VEGF levels have been found to be significantly elevated in the ocular fluids such as humor vitreous and humor aqueous of patients with proliferative diabetic retinopathy [33], and many studies confirmed its primary role in the neovascularization and in the breakdown of the blood-retinal barrier [6, 34]. We found that retinal VEGF levels increase in STZ rats and can be blunted by FE treatment (Figure 1(b)).

Oxidative stress elicited by diabetes might play a role in the development of diabetic complications [1]. Many studies have demonstrated both the crucial role of oxidative stress in the retina of diabetic animals [35] and the correlation between increased serum lipid hydroperoxides and the prevalence of retinopathy in diabetic patients [36]. Lipid peroxidation is a free radical-induced process leading to oxidative deterioration of polyunsaturated fatty acids (PUFAs). Under physiological conditions, the concentrations of plasma lipid peroxides are low. Our results show that in diabetic animals the levels of TBARS were high in the plasma and were restored to normal values after the treatment with FE.

Kowluru et al. [12] investigated the effect of long-term administration of different antioxidants on the development of retinal capillary lesions in two animal models of the early stages of diabetic retinopathy. They demonstrated in their animal model that a multiantioxidant diet that significantly inhibited (by 55–65%) the formation of both pericyte ghosts and acellular capillaries, and significantly blunted oxidative stress. Previous studies demonstrated an increase of lipid peroxide levels in the vitreous of patients suffering from proliferative diabetic retinopathy [37]. Increased serum lipid peroxides indicate that an increased free radical activity is associated with retinopathy, with pathogenetic implications [37]. It is interesting to note that, in the present study, treatment with the complex extract mixture inhibited such oxidative stress induced by the diabetic state. 

## 5. Statistical Analysis

All values are expressed as mean ± SD. The results were analyzed by one-way ANOVA followed by a Bonferroni post hoc test for multiple comparisons. Differences were considered statistically significant when *P* values were less than 0.05.

## 6. Conclusions

These data suggest that FE of red berry, GBE, and white willow bark, along with *α*-lipoic acid and L-carnosine may be useful in the treatment of diabetic retinopathy and that clinical studies to evaluate this possibility are warranted.

## Figures and Tables

**Table 1:** Effects of STZ-induced diabetes on body weight and blood glucose levels after 10 days. Control (nondiabetic) group is rats injected with only the vehicle used to dissolve STZ. FE was given intraperitoneally for 10 days. Diabetes was induced by 60 mg/kg (i.v.) injection of STZ.

Groups	Body weight (g)	Blood glucose (mg/dL)
Control	215 ± 15	98 ± 10
Diabetic	170 ± 12*	360 ± 30**
Diabetic + FE	180 ± 20*	380 ± 15**

Data are expressed as mean ± SD.

*P < 0.01, **P < 0.0001 versus control; (*n* = 8–10).

## List of Abbreviations

VEGF: Vascular endothelial growth factor
TNF-*α*: Tumor necrosis factor-alpha
ICAM-1: Intercellular adhesion molecule-1
eNOS: Endothelial nitric oxide synthase
8-OHdG: 8-Hydroxy-2′-deoxyguanosine
NF-*κ*B: Nuclear factor kappa-light-chain-enhancer of activated B cells
ROS: Reactive oxygen species
AGE: Advanced glycation end-product
RNS: Reactive nitrogen species
CN-1: Carnosinase
GBE: *Ginkgo biloba* leaf extract
TBARS: Thiobarbituric acid reactive substances
LDL: Low-density lipoprotein
STZ: Streptozotocin
ELISA: Enzyme-linked immunoSorbent assay
FE: Fortified extracts
COX-2: Cyclooxygenase enzyme-2
MDA: malonaldehyde.
